# Effects of Image Quality on the Accuracy Human Pose Estimation and Detection of Eye Lid Opening/Closing Using Openpose and DLib

**DOI:** 10.3390/jimaging8120330

**Published:** 2022-12-19

**Authors:** Run Zhou Ye, Arun Subramanian, Daniel Diedrich, Heidi Lindroth, Brian Pickering, Vitaly Herasevich

**Affiliations:** 1 Princess Margaret Cancer Centre, University Health Network, Toronto, ON M5G 2C4, Canada; 2 Division of Endocrinology, Department of Medicine, Centre de Recherche du CHUS, Sherbrooke, QC J1H 5N4, Canada; 3 Department of Anesthesiology and Perioperative Medicine, Mayo Clinic, Rochester, MN 55902, USA; 4 Department of Nursing, Mayo Clinic, Rochester, MN 55902, USA

**Keywords:** deep learning, image quality, pose estimation, facial feature extraction

## Abstract

Objective: The application of computer models in continuous patient activity monitoring using video cameras is complicated by the capture of images of varying qualities due to poor lighting conditions and lower image resolutions. Insufficient literature has assessed the effects of image resolution, color depth, noise level, and low light on the inference of eye opening and closing and body landmarks from digital images. Method: This study systematically assessed the effects of varying image resolutions (from 100 × 100 pixels to 20 × 20 pixels at an interval of 10 pixels), lighting conditions (from 42 to 2 lux with an interval of 2 lux), color-depths (from 16.7 M colors to 8 M, 1 M, 512 K, 216 K, 64 K, 8 K, 1 K, 729, 512, 343, 216, 125, 64, 27, and 8 colors), and noise levels on the accuracy and model performance in eye dimension estimation and body keypoint localization using the Dlib library and OpenPose with images from the Closed Eyes in the Wild and the COCO datasets, as well as photographs of the face captured at different light intensities. Results: The model accuracy and rate of model failure remained acceptable at an image resolution of 60 × 60 pixels, a color depth of 343 colors, a light intensity of 14 lux, and a Gaussian noise level of 4% (i.e., 4% of pixels replaced by Gaussian noise). Conclusions: The Dlib and OpenPose models failed to detect eye dimensions and body keypoints only at low image resolutions, lighting conditions, and color depths. Clinical Impact: Our established baseline threshold values will be useful for future work in the application of computer vision in continuous patient monitoring.

## 1. Introduction

Recent advances in computer vision are being applied in a number of industries including the healthcare sector. Outside of healthcare, numerous algorithms have been applied in autonomous driving [1], facial recognition in airports [2,3], self-service Amazon convenience stores [4], and cybersecurity [5]. In healthcare, those technologies are predominantly used in radiology and other imaging processing. For example, the classification of mammography using convolutional neural networks showed high sensitivity and specificity in detecting breast neoplasms [6,7,8]. Furthermore, encoder–decoder convolutional networks and cycle-consistent generative adversarial networks have also shown promise in a plethora of image semantic segmentation and translation tasks, including semantic segmentation of organs and tissues using ultrasound [9,10], MRI [11,12], and CT [13,14,15,16] images.

Another important area of application of computer vision in the healthcare setting is in continuous hospitalized patients’ activity monitoring [17,18,19]. It has been demonstrated that cameras can be installed in hospital rooms to capture continuous video feeds of patients; machine-learning algorithms can then be applied to detect body movements, facial expressions, emotions, eyelid and pupil movement, and eye-opening/closing and classify whether the patient is lying down, sitting-up, or ambulating [20]. Assessing patient eyelid opening/closing is particularly important in the context of detecting a patient’s level of consciousness (e.g., to calculate the Glasgow coma scale) and recording patient sleep/wake cycles. A priori, this type of system may serve as a detection device for early signs of patient deterioration or risks to patient safety such as falls or delirium without the need for a dedicated observer. Moreover, this new layer of information can also augment traditional patient data to aid clinical decision-making and accurate disease prognostication.

However, there may be several obstacles when applying current computer vision algorithms in the hospital setting [18,21]. First, limitations in camera placement can result in lower relative dimensions of facial and bodily features. Different camera models result in varied or lower video or image resolution and quality, which may be desirable given limited computational resources and the large numbers of patients monitored simultaneously. Furthermore, hospital room lighting is never constant, and lower levels result in darker images with lower color depth and higher noise [22,23,24,25,26,27]. The low lighting during the night can in part be circumvented by using cameras with larger sensors or infrared imaging [28].

To our knowledge, insufficient literature has assessed the effects of image resolution, color depth, noise level, and low light on the inference of eye opening and closing and body landmarks from digital images.

The aim of the present study is to test the accuracy of commonly used deep-learning models applied to different image resolutions, lighting conditions, color depths, and noise levels to establish baseline threshold values when the quality of the model drops below the accepted level of performance.

These parameters are important to establish for future work in applying computer vision in actual patient monitoring. It can be hypothesized that a degraded image should gradually decrease the model’s accuracy up to a certain threshold beyond which the model will fail completely.

Previous literature on human pose estimation and facial landmark detection is first summarized, followed by a description of the methods for decreasing image quality and testing model accuracy. Results of the effects of image resolution, color depth, noise level, and low light are then reported and discussed.

## 2. Related Works

Human pose estimation using deep learning has been the subject of intense research in recent years and has been reviewed in [29]. In general, there are two types of multi-subject pose estimation algorithms. The top-down approach first detects all human subjects in a particular scene and subsequently localizes all keypoints for each given subject. Algorithms that use such a technique include G-RMI [30], Mask-RCNN [31], MSRA [32], CPN [33], and ZoomNet [34]. By combining high- and low-resolution representations through multi-scale fusion while maintaining a high-resolution backbone, HRNet [35] and HigherHRNet [36] achieved excellent keypoint detection results.

In contrast, the bottom-up technique identifies all keypoints first and then assigns each keypoint to an individual subject. Algorithms that employ this method include DeepCut [37], DeeperCut [38], and MultiPoseNet [39]. By introducing part affinity fields, OpenPose became the most popular bottom-up algorithm [40]. The concept of part affinity fields was expanded in PifPaf through the addition of a part intensity field [41].

Facial landmark detection is closely related to pose estimation and has benefited from advancements made in human pose estimation. Algorithms used for facial landmark detection have been previously reviewed in [42]. The earliest algorithms used deformable facial mesh, which has been replaced by an ensemble of regression tree models [43] such as those included in the Dlib open-source library [44]. Since they have very high computation speeds and are easy to implement, these models have become widely used in research. More recently, algorithms used in pose estimation such as HRNet [35] have been adapted for facial landmark detection [45]. In addition, other newer methods using shape model (e.g., Dense face alignment [46]), heatmap (e.g., style-aggregated network [47], aggregation via separation [48], FAN [49], and MobileFAN [50]), and direct regression (e.g., PFLD [51], deep graph learning [52], and AnchorFace [53]) techniques have been proposed.

## 3. Materials and Methods

### 3.1. Data

Two hundred images (100 images of humans with eyes open and 100 images with eyes closed) randomly chosen from the Closed Eyes in the Wild Dataset [54] were used to assess model accuracy as image quality was gradually degraded.

To generate out-of-sample images, photographs of the primary author with eyes open and closed were captured using a 13 MP smartphone camera (Moto E XT2052-1, 13 MP, f/2.0, 1/3.1), with the height of the face occupying approximately half of the image height. Images were obtained using three 300-lumen dimmable light sources placed 150 cm in front of the face.

To test the effects of image quality on the accuracy of pose estimation, images from the COCO 2017 [55] validation dataset (https://cocodataset.org/#overview, accessed on 23 June 2022) were used. Specifically, images that depict exactly one person (Figure S6) were extracted along with body keypoint annotations (921 images).

### 3.2. Model Description

Facial landmark recognition was performed using the pretrained model in Dlib v19.24.0 (http://dlib.net/, accessed on 20 June 2022). Sixty-eight key facial landmarks were predicted by the model (see Supplementary Figure S5), where points 36 to 41 and 42 to 47 delineate the right and left palpebral fissures, respectively.

Images from the COCO body keypoint dataset were used for pose estimation with the OpenPose (https://github.com/CMU-Perceptual-Computing-Lab/openpose, accessed on 12 July 2022) pretrained model v1.7.0 [40].

### 3.3. Modifications Made

Two hundred images from the Closed Eyes in the Wild Dataset were used to assess model accuracy as image quality was gradually degraded. To generate images of different resolutions (a total of 8000 images), the original images were resized from 100 × 100 pixels to 20 × 20 pixels at an interval of 10 pixels while maintaining the aspect ratio. The image color depth was successively decreased from 16.7 M colors to 8 M, 1 M, 512 K, 216 K, 64 K, 8 K, 1 K, 729, 512, 343, 216, 125, 64, 27, and 8 colors. Gaussian noise was added by replacing randomly chosen pixels with random pixels; noise intensity was changed by varying the probability of replacing a given pixel from 0% to 10% at an interval of 1% and from 10% to 50% at an interval of 10%.

For images captured using the smartphone camera, light intensity at the level of the face was measured using a smartphone light meter application (https://play.google.com/store/apps/details?id=com.tsang.alan.lightmeter&hl=en_CA&gl=US, accessed on 15 July 2022). Images under different lighting conditions were captured by varying light across 21 intensity levels from 42 to 2 lux with an interval of 2 lux. Since the smartphone-captured images had a higher resolution (1000 × 800 pixels) than images from the Closed Eyes in the Wild Dataset (100 × 100 pixels), images of different resolutions (a total of 789 images) were generated first by resizing the original images from an image height of 1000 pixels to 100 pixels at an interval of 100 pixels and then from the 100-pixel image height to 10 pixels at an interval of 10 pixels.

For the COCO 2017 keypoint dataset, images of different quality (a total of 44,208 images) were generated. To generate images of different resolutions, the original image width (500 pixels) was decreased to 50 at an interval of 50 pixels and from 50 to 5 pixels at an interval of 5 pixels. Images with different color depths and noise levels were generated with the same quality degradation scheme as outlined above for eye open–closed inference.

### 3.4. Study Procedures

Modifications to the original images and other computations were implemented in Python 3.9. Source codes are available via: https://figshare.com/s/47540b8b79b16edec831 (accessed on 25 July 2022).

### 3.5. Measurements/Statistics

The opening and closing of the eyes were quantified using the eye aspect ratio as described in [56], which is the ratio of the vertical and horizontal dimensions of the palpebral fissure. Palpebral fissure dimensions were estimated on randomly chosen images (100 images with eyes open and 100 images with eyes closed) from the Closed Eyes in the Wild Dataset. For pose estimation, the mean absolute error (MAE) of the x and y pixel coordinates of the predicted keypoint vs. the ground truth was computed. An average MAE value was calculated for all keypoints. One-way ANOVA with multiple comparisons was carried out using images of the best quality as the comparator. An adjusted *p*-value of less than 0.05 is considered statistically significant. The rate of model failure was computed by dividing the number of images for which the models were unable to detect faces/humans by the total number of images. Statistical calculations were performed in GraphPad Prism 9 and Python 3.9.

## 4. Results

### 4.1. Eye Open-Close Inference

**Figure 1:**
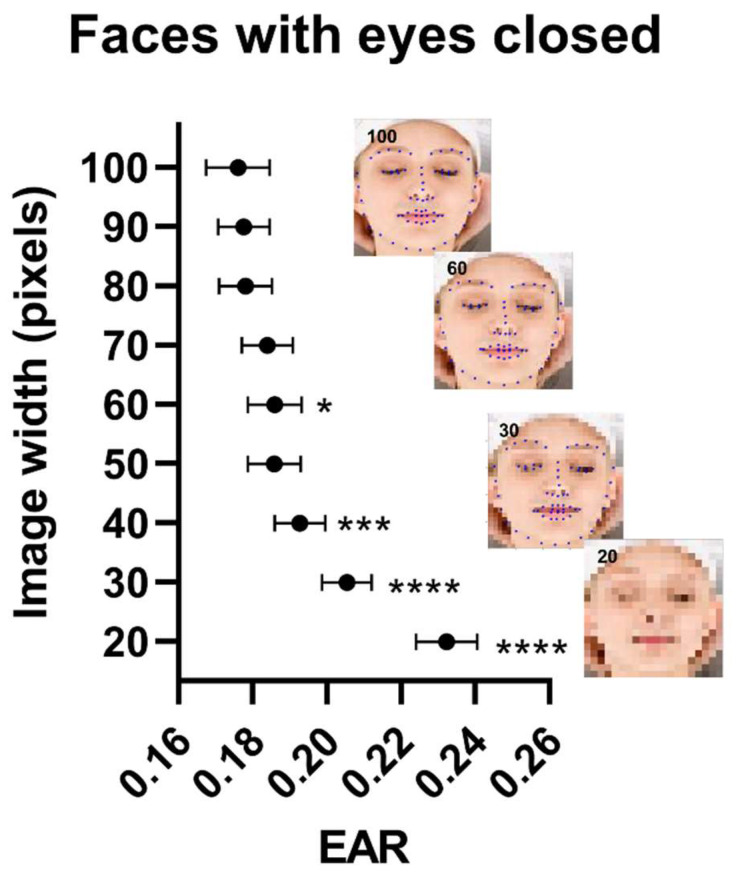
Palpebral fissure dimensions and model performance in the closed-eyes dataset as a function of image quality using the Closed Eyes in the Wild Dataset. Data points are eye aspect ratio (EAR) estimates as a function of image resolution. Inserts show images at different quality levels with overlaying model prediction. Data points ± error represent mean value ± SEM. Statistical significance levels were for one-way ANOVA with multiple comparisons using images of the best quality as the comparator. EAR: Eye aspect ratio. *: *p* < 0.05, ***: *p* < 0.001; ****: *p* < 0.0001.

As shown in Figure 1, when image resolution was reduced under 60 pixels × 60 pixels, model estimates of closed-eye dimensions (EAR of 0.19) deviated from the true dimensions (EAR of 0.18, Figure 1) and the model failed to detect the face and eyes in larger numbers of images (Figure S1D) at 30 × 30 pixels (24%) compared to the baseline (17%). Similar trends can be observed in the open-eye dataset (Figure S2A,D): EAR was 0.30 at the full image resolution (100 × 100 pixels) and deviated to 0.31 when the resolution was decreased to 50 × 50 pixels; missing values increased from 5% at baseline to 10% when the resolution was reduced to 30 × 30 pixels.

**Figure 2:**
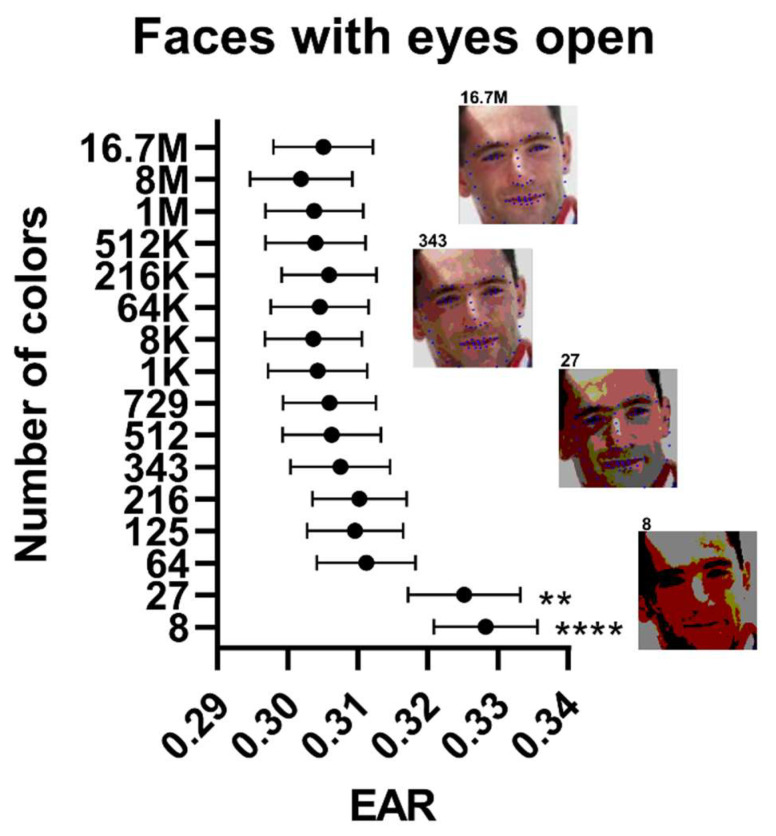
Palpebral fissure dimensions and model performance in the open-eyes dataset as a function of image quality using the Closed Eyes in the Wild Dataset. Data points are eye aspect ratio (EAR) estimates as a function of image color depth. Inserts show images at different color depths with overlaying model prediction. Data points ± error represent mean value ± SEM. Statistical significance levels were for one-way ANOVA with multiple comparisons using images of the best quality as the comparator. EAR: Eye aspect ratio. **: *p* < 0.01; ****: *p* < 0.0001.

When color depth was reduced from 16.7 M colors to 343 colors, closed-eye dimensions deviated significantly (EAR of 0.18 vs. 0.17 at baseline, Figure S1B). The deviation was highest when the color depth was reduced to 27 colors in the open (EAR of 0.33 vs. 0.30 at baseline, Figure 2) and closed (EAR of 0.19 vs. 0.17 at baseline) eye datasets. Furthermore, the percentage of missing values also increased as the color depth decreased to 343 colors (from 17% to 27% in the closed-eye dataset, Figure S1E, and from 5% to 6% in the open-eye dataset, Figure S2E).

As shown in Figures S1C and S2C, eye dimension estimates deviated from the true dimensions when 7 to 9% of the original image pixels were replaced by noise (closed-eye EAR of 0.20 with 9% noise vs. 0.17 at baseline and open-eye EAR of 0.32 with 7% noise vs. 0.30 at baseline). However, the percentage of missing values (Figures S1F and S2F) began to increase even when 4% of pixels were replaced by random noise (from 17% to 38% in the closed-eye dataset and from 5% to 10% in the open-eye dataset).

For images with different light intensities, model prediction of palpebral fissure dimension started to deviate from the true dimension as image size was reduced to image heights of 50–70 pixels (EAR of 0.20 at 1000 pixels vs. 0.22 at 70 pixels, Figure S3A, and EAR of 0.34 at 1000 pixels vs. 0.33 at 50 pixels, Figure S3C). Similarly, the number of missing values, i.e., images where the model failed to identify the face and/or both eyes, increased sharply under this image resolution: The percent of missing values increased from 19% at 40 pixels to 95% at 30 pixels in the closed-eye dataset and from 19% at 40 pixels to 76% at 30 pixels in the open-eye dataset.

**Figure 3:**
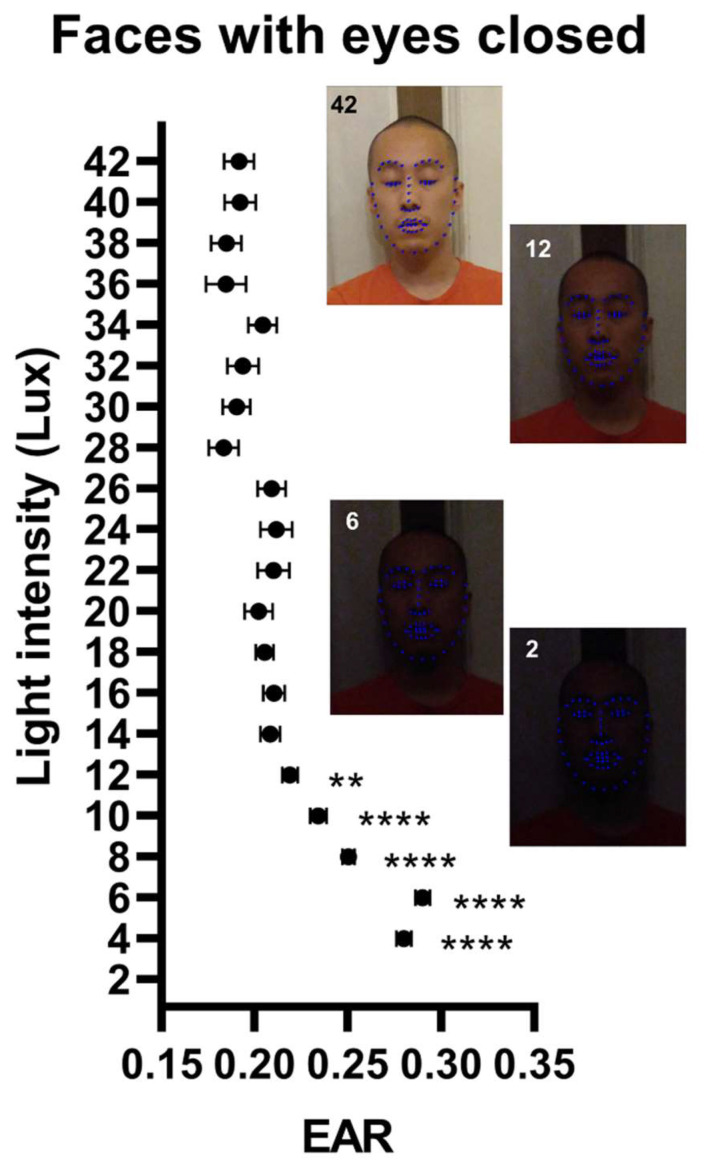
Palpebral fissure dimension and model performance as a function of image resolution and light intensity. Data points are the eye aspect ratio (EAR) estimates as a function of image lighting in faces with eyes closed. Inserts show images at different lighting levels with overlaying model prediction. Data points ± error represent mean value ± SEM. Statistical significance levels were for one-way ANOVA with multiple comparisons using images of the best quality as the comparator. EAR: Eye aspect ratio. **: *p* < 0.01; ****: *p* < 0.0001.

The model prediction of the palpebral fissure dimension deviated more gradually from the true dimension as the light intensity level decreased under 12 lux (Figure 3 and Figure S3D). At a light intensity of 8 lux, the model was increasingly less capable of correctly identifying the face and both eyes (16% at 42 lux vs. 21% at 8 lux).

### 4.2. Human Pose Estimation

**Figure 4:**
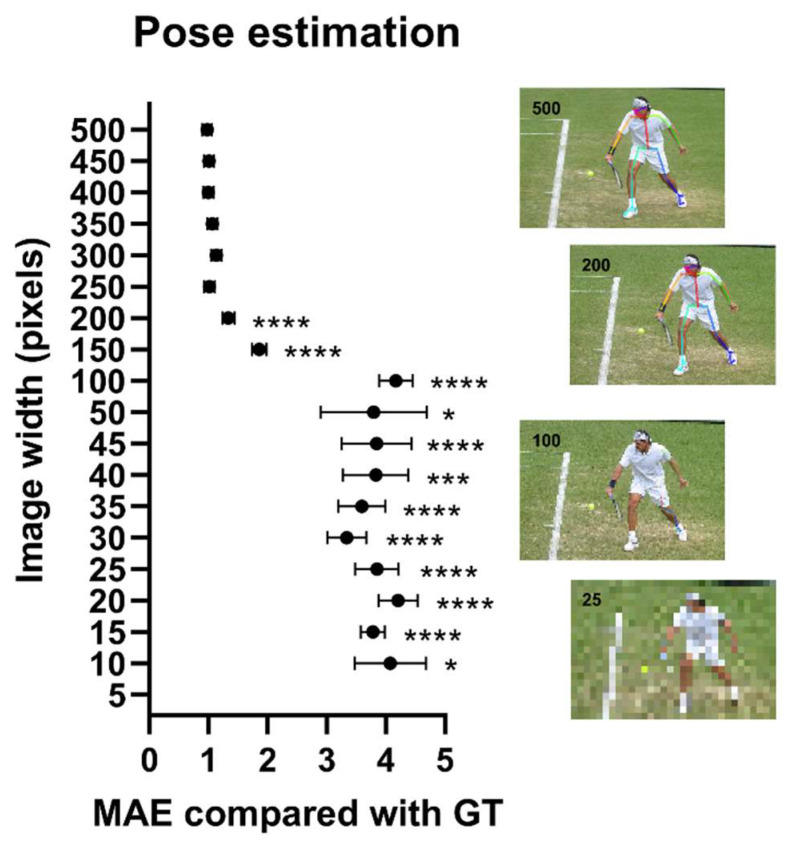
Model performance in the COCO body keypoint dataset as a function of image quality. Data points are mean absolute error values of model prediction as a function of image resolution. Inserts show images at different quality levels with overlaying model prediction. Data points ± error represent mean value ± SEM. Statistical significance levels were for one-way ANOVA with multiple comparisons using images of the best quality as the comparator. EAR: Eye aspect ratio; GT: Ground truth; MAE: Mean absolute error. *: *p* < 0.05, ***: *p* < 0.001; ****: *p* < 0.0001.

The prediction accuracy of the model for human poses using the COCO dataset decreased significantly when the image height was reduced to less than 200 of the original 500 pixels (MAE of 1.3 pixels vs. 0.98 pixels, respectively, Figure 4). Since human subjects occupied, on average, 150 × 200 pixels of the original images, this indicates that the model was accurate up to a resolution of 60 × 80 pixels that depict only the human subject. Similarly, the fraction of images where the model was unable to identify the human subject started to increase dramatically beyond this resolution threshold (the percent of missing values increased from 17% at 200 pixels to 84% at 100 pixels, Figure S4D).

When color depth was reduced to values lower than 512 colors, pose estimation began to deviate significantly from the ground truth (MAE of 0.98 pixels at 16.7 M colors vs. 1.12 pixels at 512 colors). The percentage of missing values also increased sharply as color depth was inferior to 343 colors (10% at 16.7 M colors vs. 14% at 343 colors, Figure S4E).

As shown in Figure S4C, the error of pose estimation from ground truth began to rise significantly compared to the baseline when 5% of the original image pixels were replaced by noise (0.97 pixels at baseline vs. 1.17 pixels with 5% noise). Similarly, the percentage of missing values (Figure S4F) started to increase when 4% of pixels were replaced by random noise (10% at baseline vs. 13% with 4% noise).

## 5. Discussion

This study systematically tested the effects of image quality on facial feature extraction and human pose estimation using common deep learning models.

For the determination of eye opening and closing with Dlib, the resolution of facial images can be reduced to 60 × 60 pixels without significantly affecting the model estimation of eye dimension. When the color depth of images was lower than 343 colors, eye dimensions estimated by the model began to deviate from the true eye dimensions, and it became increasingly difficult for the model to identify the face. The accuracy of model estimation of eye dimensions began to decrease when 7% of the original image pixels were replaced by noise. Interestingly, even when images of the face were taken under low lighting conditions (14 lux), eye dimensions could still be accurately determined to differentiate between open vs. closed eyes. Under very low lighting (6 lux), the model could still identify the face in most instances.

For human pose estimation using OpenPose, the resolution of regions representing human subjects can be reduced to 60 × 80 pixels without significantly affecting model accuracy or performance. Color depth reduction from 16.7 M to 512 colors resulted in a significant increase in the mean absolute error of model prediction. The addition of more than 4% Gaussian noise also increased model error.

Typically, contemporary convolutional neural networks are trained using images with resolutions greater than a few hundred pixels in width and height. Large image datasets (e.g., Microsoft COCO [55], ImageNet [57], the MPII Human Pose Dataset [58], and the CMU Panoptic Dataset [59]) used for the recognition and pose estimation of human subjects usually contain images with decent resolutions of 300 to 500 pixels in height and width. Images of similar resolutions are also contained in frequently used datasets for facial landmark annotation (e.g., the AFLW Dataset [60] and 300 W [61]) and emotion detection (e.g., AffectNet [62], CK+ [63], and EMOTIC [64]). Furthermore, in medical imaging with MRI [65,66], PET [65,67], and CT [14,68], deep learning applications are typically trained using images with resolutions ranging from 128 × 128 to 512 × 512 pixels.

Previous studies have investigated the application of pose estimation algorithms in low-resolution images [69,70]. However, insufficient literature has assessed the effects of image resolution, color depth, noise level, and low light on the inference of eye opening and closing and body landmarks from digital images. Therefore, in the present study, the accuracy of commonly used deep-learning models while varying image resolutions, lighting conditions, color depths, and noise levels was tested. This allowed us to establish baseline threshold values for future work applying computer vision in continuous patient monitoring.

Limitations of this work include the use of relatively small datasets of images; therefore, our study may be underpowered to detect changes in model prediction with small decreases in image quality. Furthermore, subjects in the COCO body keypoint dataset do not all occupy the same number of pixels, which may have introduced heterogeneity in model accuracy. Future work may therefore be performed by testing multiple different networks for a given task using larger numbers of images. In addition, variability (e.g., head tilt) exists in the photos captured by the smartphone camera, which may be an obstacle to the reproducibility of the results. In addition, only the OpenPose and DLib models were tested without model finetuning; other newer deep learning models (e.g., Retinaface [71] and Mediapipe [72,73]) should be studied in future works. Future works should also assess the effects of video instead of photo quality on model accuracy.

## 6. Conclusions

In this study, the effects of image quality on facial feature extraction and human pose estimation using the Dlib and OpenPose models were systematically assessed. It is found that, so far, these models only failed to detect eye dimensions and body keypoints at very low image resolutions (the failure rate for eye dimension estimation increased from below 20% to over 70% by decreasing the facial image resolution from 40 × 40 to 30 × 30 pixels), lighting conditions (the failure rate for eye dimension estimation of 16% at 42 lux light intensity vs. 21% at 8 lux), and color depths (failure rate for pose estimation of 10% at 16.7 M colors vs. 14% at 343 colors). Our established baseline threshold values will be essential for future work in the application of computer vision in continuous patient monitoring.

## Data Availability

Modifications to the original images and other computations were implemented in Python 3.9. Source codes are available at https://drive.google.com/drive/folders/1XnI62_b_cgTZV1VnNpxmx74OwTFy_yCk?usp=sharing (accessed on 28 July 2022).

## Supplementary Materials

The following supporting information can be downloaded at: https://www.mdpi.com/article/10.3390/jimaging8120330/s1, Supplementary material. Supplementary Figure S1. Palpebral fissure dimension and model performance in the closed-eyes dataset as a function of image quality using the Closed Eyes in the Wild Dataset. Supplementary Figure S2. Palpebral fissure dimension and model performance in the open-eyes dataset as a function of image quality using the Closed Eyes in the Wild Dataset. Supplementary Figure S3. Palpebral fissure dimension and model performance as a function of image resolution and light intensity. Supplementary Figure S4. Model performance in the COCO body keypoint dataset as a function of image quality. Supplementary Figure S5. Facial landmarks prediction using Dlib showing an example of model output with the localization of 64 landmarks.
