# Supplementary material for: Effects of Image Quality on the Accuracy Human Pose Estimation and Detection of Eye Lid Opening/Closing Using Openpose and DLib
Source: J Imaging. 2022 Dec 19;8(12):330. doi: 10.3390/jimaging8120330 (PMC9783075; doi:10.3390/jimaging8120330)
Supplement: Supplementary file 1 [file jimaging-08-00330-s001.zip › jimaging-1998426-supplementary.pdf]

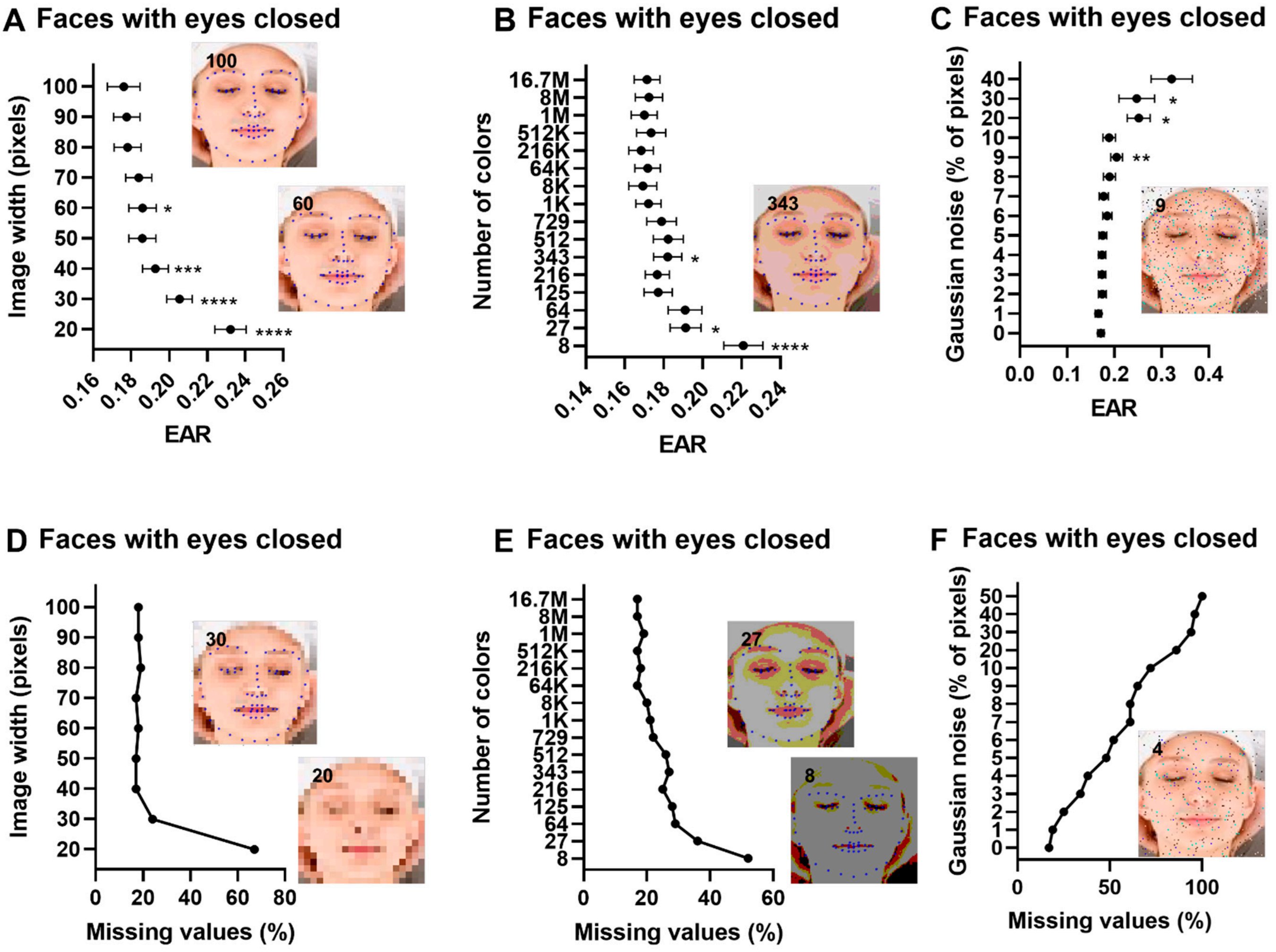

Supplementary Figure S1. Palpebral fissure dimension and model performance in the closed-eyes dataset as a function of image quality using the Closed Eyes in the Wild Dataset. Panels (A) to (C) show the eye aspect ratio (EAR) estimates as a function of image resolution, color depth, and gaussian noise, respectively. Panels (D) to (F) show the percentage of missing values where the model failed to identify the face and/or both eyes as a function of image resolution, color depth, and gaussian noise, respectively. Inserts show images at different quality levels with overlaying model prediction. Data points ± error represent mean value ± SEM. Statistical significance levels were for one-way ANOVA with multiple comparisons using images of the best quality as the comparator. EAR: eye aspect ratio. *: P < 0.05, **: P < 0.01; ***: P < 0.001; ***: P < 0.0001

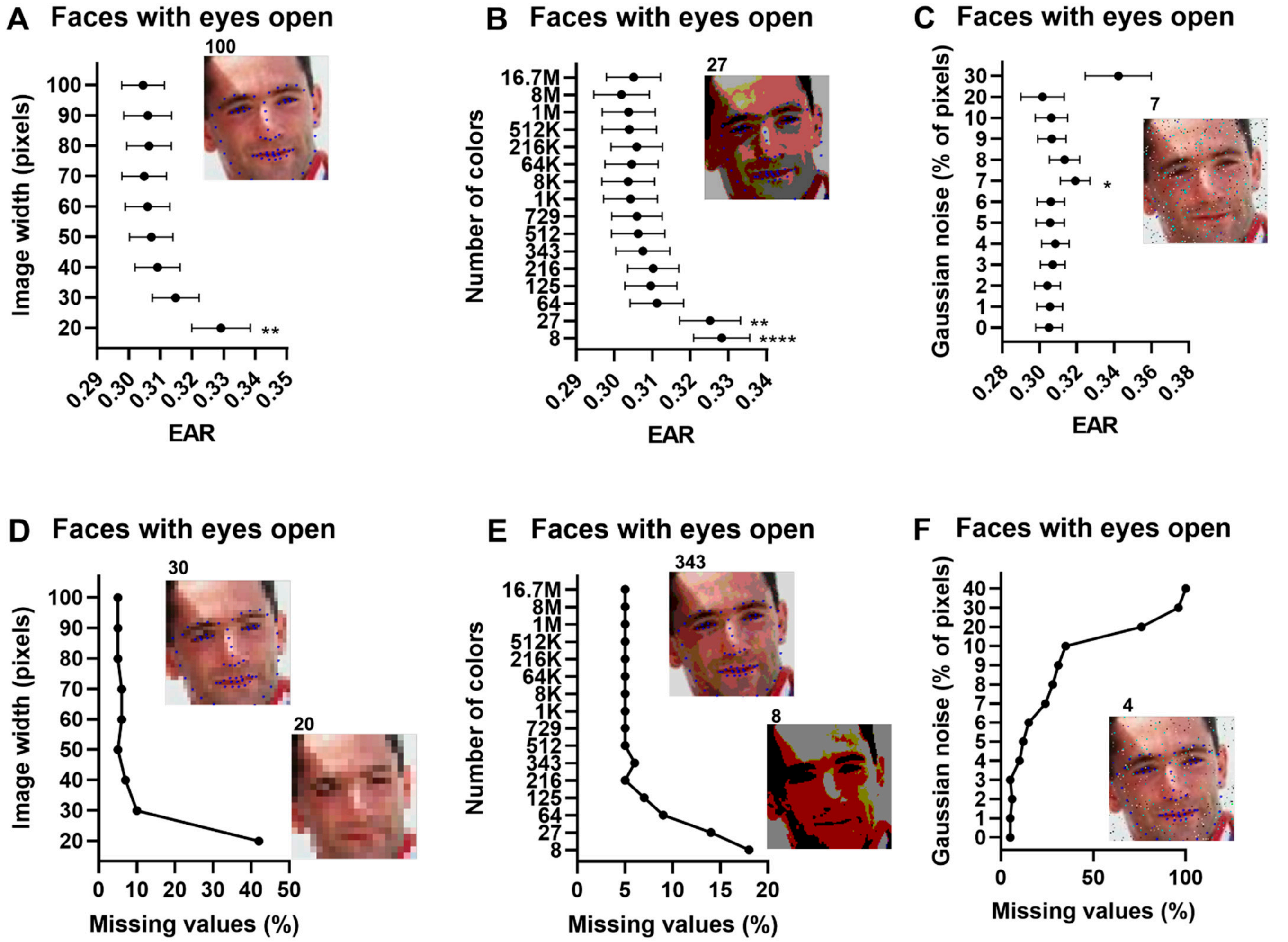

Supplementary Figure S2. Palpebral fissure dimension and model performance in the open-eyes dataset as a function of image quality using the Closed Eyes in the Wild Dataset. Panels (A) to (C) show the eye aspect ratio (EAR) estimates as a function of image resolution, color depth, and gaussian noise, respectively. Panels (D) to (F) show the percentage of missing values where the model failed to identify the face and/or both eyes as a function of image resolution, color depth, and gaussian noise, respectively. Inserts show images at different quality levels with overlaying model prediction. Data points ± error represent mean value ± SEM. Statistical significance levels were for one-way ANOVA with multiple comparisons using images of the best quality as the comparator. EAR: eye aspect ratio. *: $P < 0.05$, **: $P < 0.01$; ***: $P < 0.001$; ***: $P < 0.0001$

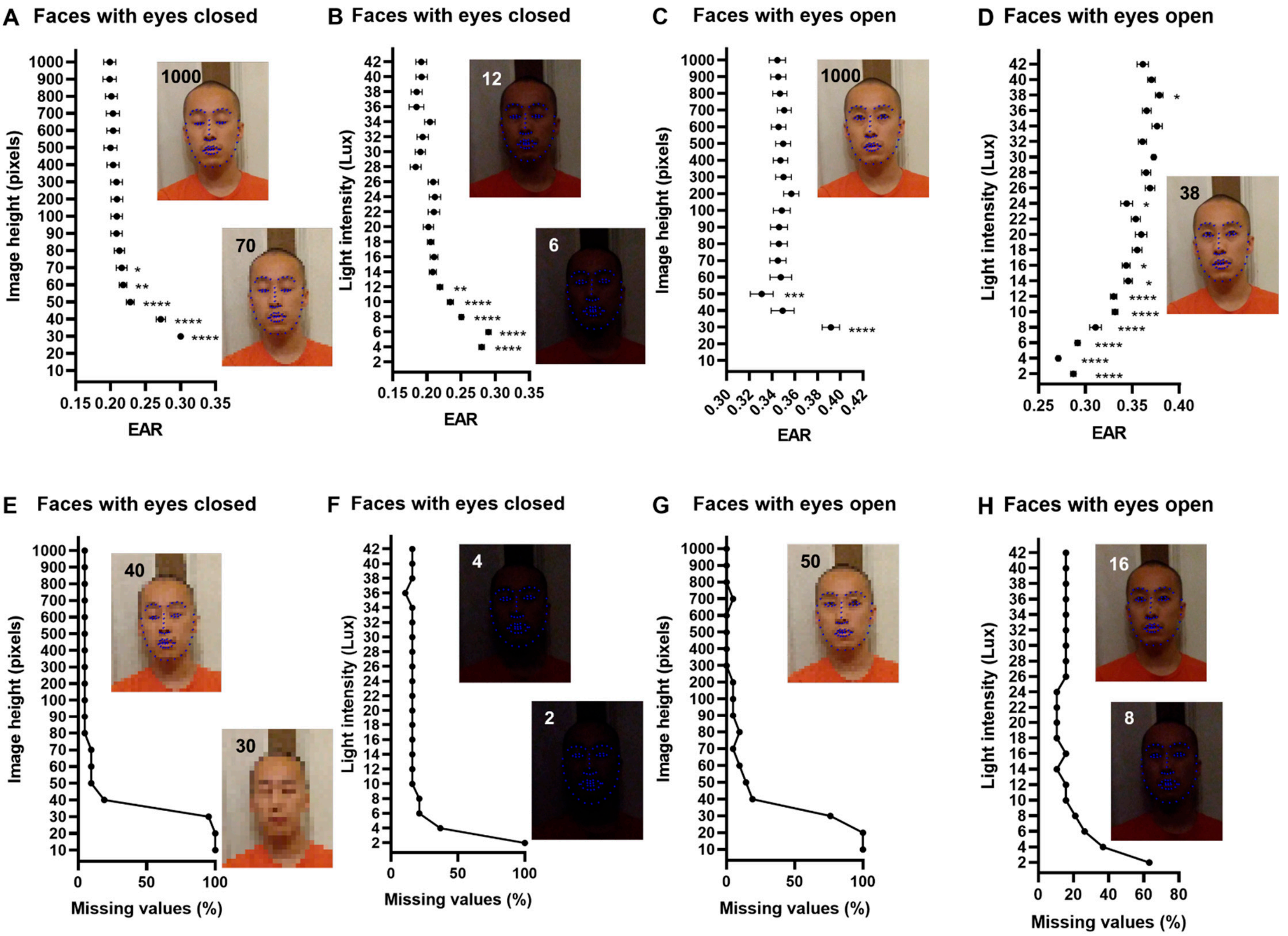

Supplementary Figure S3. Palpebral fissure dimension and model performance as a function of image resolution and light intensity. Panels (A) and (B) show the eye aspect ratio (EAR) estimates as a function of image resolution in faces with eyes closed. Panels (C) and (D) show the same in faces with eyes open. Panels (E) to (F) show the percentage of missing values where the model failed to identify the face and/or both eyes as a function of image resolution in faces with eyes closed. Panels (G) and (H) show the same in faces with eyes open. Inserts show images at different quality levels with overlaying model prediction. Data points ± error represent mean value ± SEM. Statistical significance levels were for one-way ANOVA with multiple comparisons using images of the best quality as the comparator. EAR: eye aspect ratio. *: $P < 0.05$, **: $P < 0.01$; ***: $P < 0.001$; ***: $P < 0.0001$

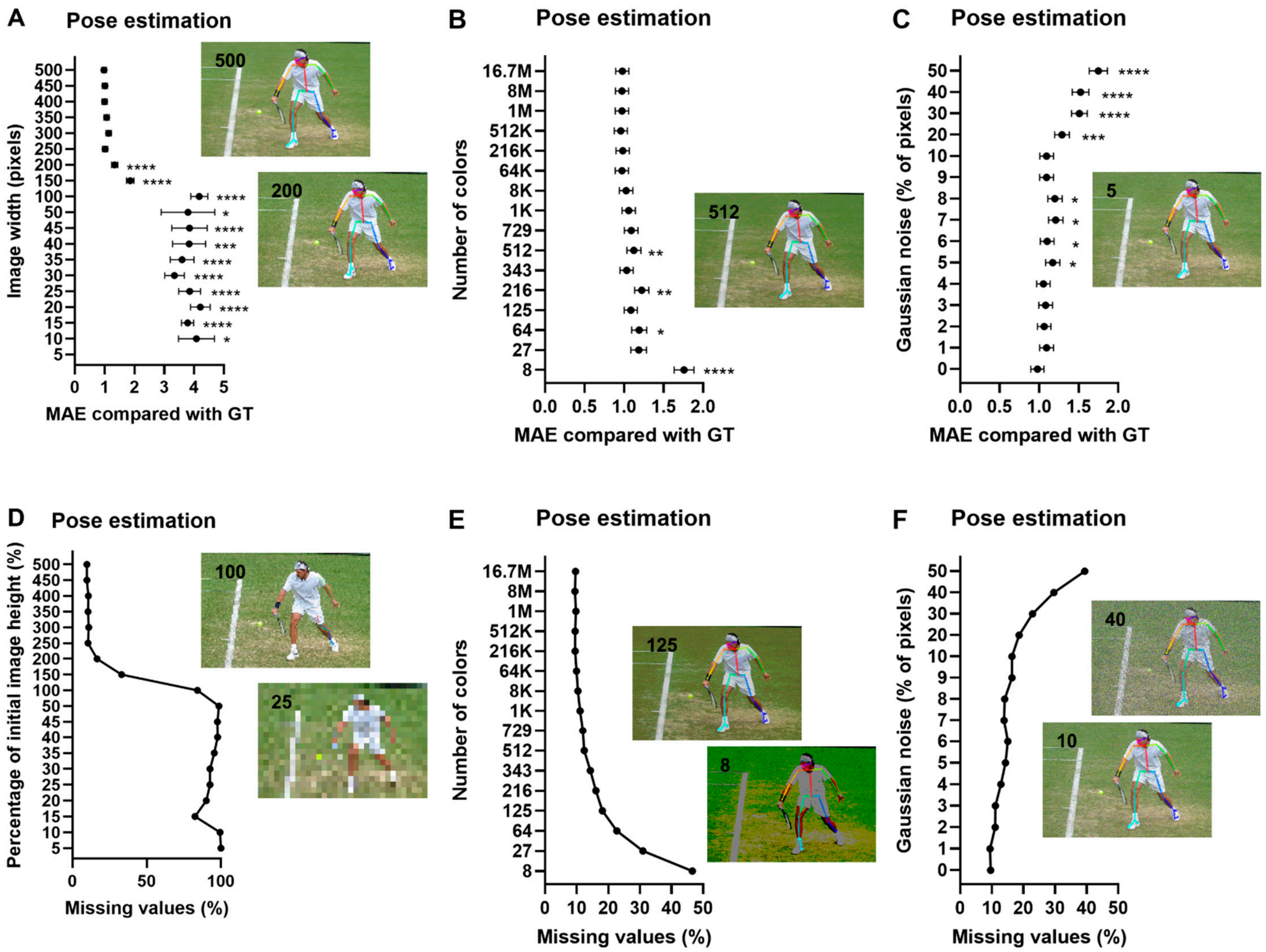

Supplementary Figure S4. Model performance in the COCO body keypoint dataset as a function of image quality. Panels (A) to (C) show the mean absolute error of model prediction as a function of image resolution, color depth, and gaussian noise, respectively. Panels (D) to (F) show the percentage of missing values where the model failed to identify the body as a function of image resolution, color depth, and gaussian noise, respectively. Inserts show images at different quality levels with overlaying model prediction. Data points ± error represent mean value ± SEM. Statistical significance levels were for one-way ANOVA with multiple comparisons using images of the best quality as the comparator. EAR: eye aspect ratio; MAE: mean absolute error. *: P < 0.05, **: P < 0.01; ***: P < 0.001; ***: P < 0.0001

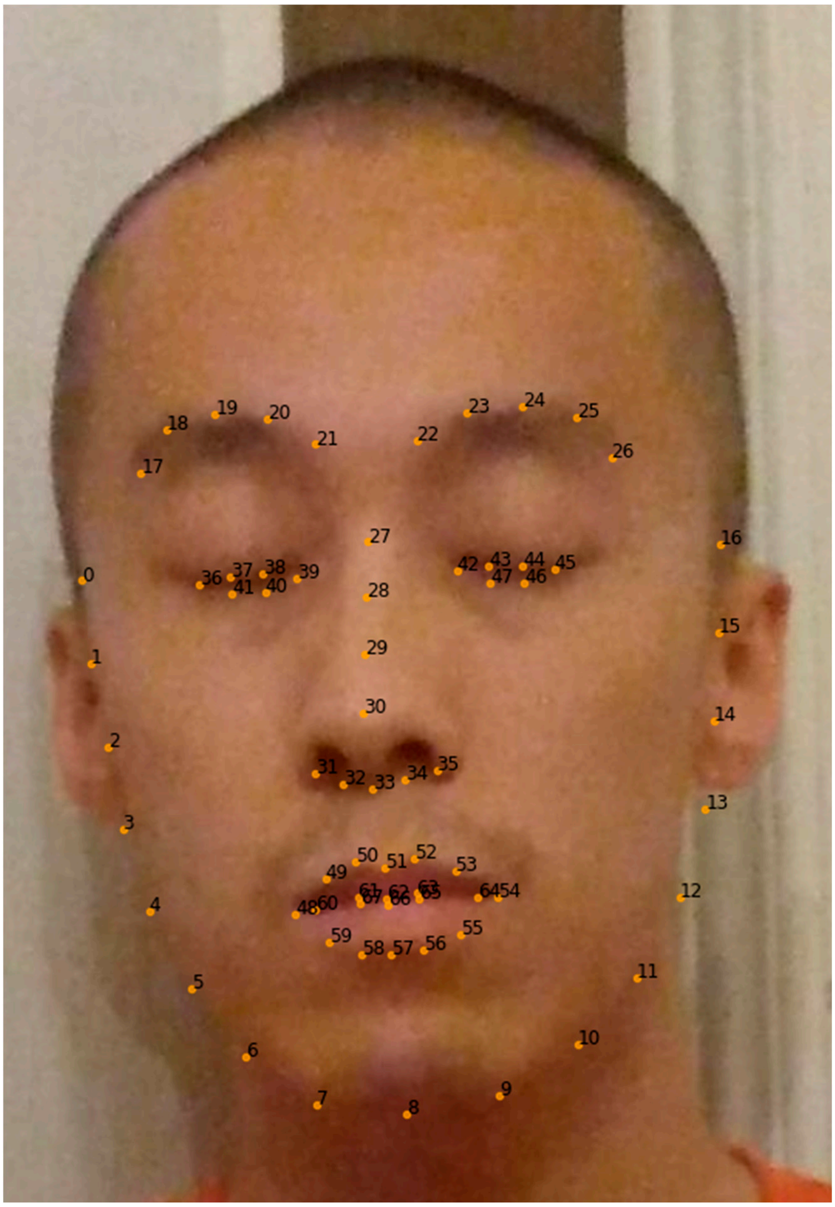

Supplementary Figure S5. Facial landmarks prediction using Dlib showing an example of model output with the localization of 64 landmarks.
